# Diagnostic value of echocardiographic markers for diastolic dysfunction and heart failure with preserved ejection fraction

**DOI:** 10.1007/s10741-020-09985-1

**Published:** 2020-06-02

**Authors:** Elisa Dal Canto, Sharon Remmelzwaal, Adriana Johanne van Ballegooijen, M. Louis Handoko, Stephane Heymans, Vanessa van Empel, Walter J. Paulus, Giel Nijpels, Petra Elders, Joline WJ Beulens

**Affiliations:** 1 Department of Epidemiology and Biostatistics, Amsterdam University Medical Center, Amsterdam, The Netherlands; 2 Department of General Practice and Elderly Care Medicine, Amsterdam University Medical Center, Amsterdam, The Netherlands; 3 Department of Nephrology, Amsterdam University Medical Center, Amsterdam, The Netherlands; 4 Department of Cardiology, Amsterdam University Medical Center, Amsterdam, The Netherlands; 5 Department of Cardiology, CARIM School for Cardiovascular Diseases, Maastricht University Medical Centre, Maastricht, The Netherlands; 6 Department of Cardiovascular Sciences, Centre for Molecular and Vascular Biology, Leuven, KU Belgium; 7 The Netherlands Heart Institute (Nl-HI), Utrecht, The Netherlands; 8 Department of Physiology, Amsterdam University Medical Center, Amsterdam, The Netherlands; 9 Julius Center for Health Sciences and Primary Care, Utrecht University Medical Center, Utrecht, The Netherlands

**Keywords:** Heart failure with preserved ejection fraction, Diastolic dysfunction, Echocardiography, Systematic review, Meta-analysis

## Abstract

This study aimed to evaluate the diagnostic performance of echocardiographic markers of heart failure with preserved ejection fraction (HFpEF) and left ventricular diastolic dysfunction (LVDD) in comparison with the gold standard of cardiac catheterization. Diagnosing HFpEF is challenging, as symptoms are non-specific and often absent at rest. A clear need exists for sensitive echocardiographic markers to diagnose HFpEF. We systematically searched for studies testing the diagnostic value of novel echocardiographic markers for HFpEF and LVDD. Two investigators independently reviewed the studies and assessed the risk of bias. Results were meta-analysed when four or more studies reported a similar diagnostic measure. Of 353 studies, 20 fulfilled the eligibility criteria. The risk of bias was high especially in the patients’ selection domain. The highest diagnostic performance was demonstrated by a multivariable model combining echocardiographic, clinical and arterial function markers with an area under the curve of 0.95 (95% CI, 0.89–0.98). A meta-analysis of four studies indicated a reasonable diagnostic performance for left atrial strain with an AUC of 0.83 (0.70–0.95), a specificity of 93% (95% CI, 90–97%) and a sensitivity of 77% (95% CI, 59–96%). Moreover, the addition of exercise E/e′ improved the sensitivity of HFpEF diagnostic algorithms up to 90%, compared with 60 and 34% of guidelines alone. Despite the heterogeneity of the included studies, this review supported the current multivariable-based approach for the diagnosis of HFpEF and LVDD and showed a potential diagnostic role for exercise echocardiography and left atrial strain. Larger well-designed studies are needed to evaluate the incremental value of novel diagnostic tools to current diagnostic algorithms.

**Electronic supplementary material:**

The online version of this article (10.1007/s10741-020-09985-1) contains supplementary material, which is available to authorized users.

## Introduction

Heart failure with preserved ejection fraction (HFpEF) is a complex clinical syndrome associated with high morbidity and mortality, which now accounts for 56% of the subjects with HF, and its prevalence is increasing [1]. HFpEF is defined by the presence of symptoms and/or signs of HF, a preserved left ventricular (LV) ejection fraction (LVEF, > 50%), elevated levels of natriuretic peptides (NPs) and the evidence of cardiac functional and structural alterations underlying HF [2]. Structural alterations include an increased left atrial volume index (LAVI) or left ventricular mass index (LVMI), whereas functional alterations mostly include left ventricular diastolic dysfunction (LVDD). LVDD is defined as the presence of impaired LV relaxation and increased LV chamber stiffness, which increases LV filling pressures (LVFP) [3]. Evidence of LVDD can be obtained invasively through rest or exercise right-sided heart catheterization or non-invasively through echocardiography [2]. There is no single echocardiographic measure that provides evidence of LVDD, but rather a combination of several abnormal indices is recommended to evaluate LV diastolic function: tissue Doppler indices (E/e′ ratio and e’ velocities), LAVI and tricuspid regurgitation velocity are the currently recommended variables [3]. However, only a relatively small number of studies validated the use of these echocardiographic indices, showing only a modest correlation with invasive haemodynamic parameters and limited discriminative power [4]. Additionally, the echocardiographic indices proposed by guidelines are normal in 40–75% of subjects with invasively proven HFpEF [4, 5] and showed lower accuracy in individuals at an early stage of the disease. In fact, these subjects often show a normal or indeterminate diastolic function at resting echocardiography because LVFP are not elevated, or because they vary over time, depending on volume status [6]. Recently, a new stepwise diagnostic approach that includes clinical, laboratory and imaging tests—the HFA-PEFF score—was proposed by the Heart Failure Association (HFA) of the European Society of Cardiology (ESC) with the purpose of integrating novel information into a comprehensive algorithm, in order to better identify subjects with HFpEF at different stages [7]. These recommendations include some of the new techniques that are currently being evaluated as potential diagnostic tools to improve diagnosis and staging of subjects with HFpEF, such as measures of LV deformation by 2D speckle tracking echocardiography (STE) and diastolic stress test (DST)-derived parameters [7]. In addition to these, left atrial (LA) functional parameters such as LA strain recently demonstrated significant correlation with both clinical status and invasive measures of LVFP in subjects with HFpEF and thus could improve HFpEF diagnosis [8]. With this review, we aim to systematically evaluate the diagnostic value of novel echocardiographic indices and multivariable models on accuracy and incremental utility to identify LVDD and HFpEF.

## Methods

### Data sources and searchers

We performed a systematic review of PubMed and EMBASE from their inception to (SR and LS) May 13, 2019, according to the PRISMA-DTA Statement [8]. Search terms included indexed terms from MeSH in PubMed and EMBASE, as well as free-text terms. This search was used for a set of three systematic reviews that describe different types of diagnostic markers for LVDD and HFpEF (NPs, echocardiographic markers and biomarkers). Bibliographies of the identified articles were also hand-searched for relevant publications (see Appendix A). The protocol and search strategy was preregistered on PROSPERO (registration number: CRD42018065018).

### Study selection

Two reviewers independently screened titles, abstracts and full-text articles. Inconsistencies in study selection were resolved through discussion until consensus was reached and, if needed, through the consultation of a third reviewer (SR, EDC, AJvB or JWJB). The eligibility of studies was assessed according to the inclusion and exclusion criteria listed in Supplementary Table 1.

### Quality assessment

Two reviewers (SR and EDC) independently performed the quality assessment for each study using the QUADAS-2 tool (Quality Assessment of Diagnostic Accuracy Studies) [9]. QUADAS-2 consists of four domains: patient selection, index test, reference standard and flow and timing. Quality is assessed in each domain to estimate risk of bias and concerns regarding applicability. The patient selection domain assessed whether the selection of participants could have introduced bias. The index test and reference standard domains assessed whether the conduct or interpretation of the index test and reference standard, respectively, may have introduced bias. The flow and timing domain addressed the time interval between index test and reference standard (9). Any discrepancies or disagreements between the authors were resolved through discussion until consensus was reached and, if needed, through the consultation of a third reviewer (JWJB).

### Diagnostic performance and data extraction

Two authors (SR and EDC) extracted data independently, according to a standard protocol that included first author, year of publication, country, journal, study design, markers (echocardiographic ± clinical/laboratory parameters), outcome measures, population description, reference diagnosis and measures of diagnostic performance.

### Data synthesis

Study characteristics of the studies were described in a systematic manner according to the diagnostic markers. Studies were meta-analysed using a random-effects model when three or more studies investigated the same diagnostic measure for the same echocardiographic marker in a similar study population and with a similar control population. In addition, the studies had to provide confidence intervals (95% CI) of this diagnostic performance measure or sufficient information (2 × 2 table) to compute these confidence intervals. Forest plots of random-effects meta-analyses were fitted for AUCs, sensitivities and specificities. Heterogeneity was tested using *I*^2^, where an *I*^2^ > 75% is considered as substantial heterogeneity. All analyses and plots were performed in RStudio version 3.4.2 using the metafor package [10].

## Results

### Search results

We screened 11,727 titles, which yielded 353 potentially relevant studies. In total, 20 studies met the inclusion criteria. The remainder was excluded according to the criteria listed in the PRISMA flowchart (see Supplementary Fig. 1).

### Quality assessment

The QUADAS-2 domain with the highest proportion of high risk of bias was patient selection (Supplementary Fig. 2) with 13 studies (65%) demonstrating a high risk of bias mostly due to case-control design or to a non-consecutive or non-random inclusion of subjects. In the other three of the four QUADAS-2 domains (index test, reference test and flow and timing), eight (40%), three (15%) and eight (40%) studies, respectively, demonstrated a high of risk of bias. In the QUADAS-2 domain reference standard, 12 studies (60%) showed an unclear risk of bias. On the other hand, most of the studies showed low concerns regarding applicability with the highest proportion of high concerns for the reference standard domain (six studies, 30%). None of the studies was excluded based on the quality assessment.

### Study characteristics

Of the 20 included studies, ten were performed in the USA, seven in Europe, two in Japan and one in Australia (Table 1). Eighteen studies (90%) were published in the past 10 years (2009–2019). Sixteen were cross-sectional and four were case-control studies performed in subjects referred for right- and/or left-sided heart catheterization. As clinical outcome, 13 studies used HFpEF [5, 11–22], two used HFpEF with associated pulmonary hypertension (PH)  [23, 24], one used “early” HFpEF [25], and four used LVDD  [26–29]. The reference diagnosis always included the echocardiographic evidence of a normal LVEF and one or more invasive measures of elevated LVFP (LV end-diastolic pressure or pulmonary capillary wedge pressure), impaired LV relaxation (isovolumetric relaxation time or constant τ) and increased LV stiffness (LV stiffness constant *b*). Conventional transthoracic rest echocardiography was the most commonly used index measure (*n* = 10) followed by STE (*n* = 8) and by DST (*n* = 2). As echocardiographic predictor, seven studies used a combination of echocardiographic markers or multivariable models that included also demographics, medications, biochemical and arterial function parameters; eight studies used LV and LA strain parameters; and two studies used DST data and three of them used single standard echocardiographic parameters.

**Table 1:** Baseline characteristics of the 20 included studies

Study	Study design	Predictors	Outcome	Study population	Index group (*n*); sex (% female); age	Reference group (*n*)	Reference diagnosis
HFpEF studies							
Multivariable models and echocardiographic equations							
Thenappan USA [23]	Cross-sectional	Age, clinical data, echo, haemodynamics	PH-HFpEF	PH registry	PH-HFpEF (100); 82%; 64 ± 13	PAH (522)	HF symptoms + LVEF > 50% + PWCP > 15 mmHg/LVEDP > 15 mmHg/PVR > 2.5 wood units
Weber EU [11]	Cross-sectional	E/e′ + other echo, arterial function, clinical data	HFpEF	Subjects referred to RHC for suspected CAD	HFpEF (71); 33.8%; 67.7 ± 8.6	Non-HFpEF (65)	LVEF > 50% + LVEDP > 16 mmHg + NT-proBNP > 220 pg/mL
Cameron USA [24]	Cross-sectional	2009 ASE/EAE guidelines + multivariable models	PH-HFpEF	Subjects enrolled in the PH program for the assessment of PH	PH + LVED > 15 mmHg (81); 67%; 62 (56–70)	PH + LVEDP ≤ 15 mmHg (80)	PASP > 25 mmHg + LVEDP > 15 mmHg
Dokainish USA [17]	Cross-sectional	Echocardiographic equations	HFpEF	Subjects referred to LHC for clinical reasons	LVEF > 50% + LVEDP > 20 mmHg (69); 58%; 55.1 ± 8.5	LVEF > 50% + LVEDP < 20 mmHg (53)	LVEF > 50% + LVEDP > 20 mmHg
Dini EU [16]	Cross-sectional	Echocardiographic equations	HFpEF	HF subjects	HFpEF (55); 35%; 67 ± 12	HFrEF (123)	LVEF > 50% + PCWP > 15 mmHg
Reddy USA [18]	Cross-sectional	H_2_FPEF score	HFpEF	Subjects undergoing RHC for the evaluation of dyspnoea	HFpEF (267); 61%, 68 ± 11	NCD (147); 59%, 56 ± 15	LVEF > 50%, dyspnoea + PCWP at rest ≥ 15 mmHg or during exercise ≥ 25 mmHg
Left ventricular strain and strain rate							
Kasner EU [12]	Case-control	Global strain rates and their ratios with early transmitral flow	HFpEF		HFpEF (21); 52%; 43–60	Subjects with chest pain (12)	τ ≥ 48 ms and/or LVEDP ≥ 16 mmHg and/or stiffness constant β ≥ 0.015 mL/1 and/or stiffness b ≥ 0.19 mmHg/mL + HF symptoms + normal LVEF
Wang USA [14]	Case-control	Global longitudinal strain	HFpEF		DHF (20); 35%; 63 ± 11	Healthy subjects (17)	LVEF > 50% + PCWP > 12 mmHg
Left atrial strain							
Kurt USA [13]	Case-control	E/E′/LA systolic strain (LA non-invasive stiffness)	HFpEF (DHF)		DHF (20);30%; 58 ± 16	LVH + normal LVEF (19)	Clinical criteria + PCWP (ESC 2007 guidelines)
Lundberg EU [20]	Cross-sectional	LA global strain (LA-GS), TR Vmax, LAVi and E/e′	HFpEF	Subjects referred to RHF for suspected HF	EF ≥ 50% (63) + abnormal LAP	Normal LAP (29)	Pulmonary artery wedge pressure (PAWPM) > 15 mmHg at rest or ≥ 23 mmHg during peak exercise
Reddy USA [19]	Cross-sectional	LA reservoir, conduit and booster strain, LA reservoir strain/E/e′, LA reservoir strain/LAVI	HFpEF	Subjects undergoing RHC for dyspnoea	HFpEF (238), 62%, 68 ± 10	NCD (125), 56%, 58 ± 14	Clinical symptoms of HF + LVEF ≥ 50% + PCWP with rest ≥ 15 mmHg and/or exercise ≥ 25 mmHg
Singh USA [21]	Cross-sectional	Peak LA strain	HFpEF	Subjects referred to LHC for various reasons (chest pain, ACS, etc.)	HFpEF (7)	LVDP <15 mmHg (25)	Pre-A-wave LVDP > 15 mmHg
Telles AU [22]	Cross-sectional	LA global reservoir and LA pump strain	HFpEF	Subject referred to RHC for exertional dyspnoea	HFpEF (49), 71.4%, 69.4 ± 8.0	NCD (22), 77.3%, 67.0 ± 9.9	LVEF > 50%, dyspnoea + PCWP ≥ 15 mmHg at rest and/or ≥ 25 mmHg at maximal exertion
Diastolic stress test markers							
Hammoudi EU [25]	Cross-sectional	Lateral and septal E/E′ at low-level exercise (25 and 50 W)	Early HFpEF	Subjects at high risk for HFpEF	LVEDP > 16 mmHg during exercise (34);23%, 64.8(55.2–73.4)	LVEDP < 16 mmHh (12)	LVEDP >16 mmHg
Obokata USA [5]	Cross-sectional	ESC algorithm + exercise average E/E′	HFpEF	Subjects referred to RHC for exertional dyspnoea	HFpEF (50); 54%; 70 ± 11	NCD (24)	HF symptoms, LVEF ≥ 50%, PCWP at rest > 15 mmHg and/or with exercise ≥ 25 mmHg
Single conventional echocardiography markers							
Nagueh USA [15]	Cross-sectional	Echo estimated RAP > 8 mmHg	HFpEF	Subjects with exertional dyspnoea enrolled in a multicentre study	HFpEF (50); 44%; 64 ± 9	Non-HFpEF (79)	LVEF > 50% + PCWP > 12 mmHg
Left ventricular diastolic dysfunction studies							
Goto Jap [27]	Cross-sectional	BNP > 22.4 pg/mL + E velocity < 7.4 cm/s	LVDD	Subjects referred to LHC for the evaluation of CAD	Isolated LVDD (91); 18.7%; 67.4 ± 8.2	Normal diastolic function (189)	LVEF ≥ 50% + τ ≥ 48 ms
Weber EU [29]	Case-control	LVETI, E/A, E′ and E/E′	LVDD	Subjects with suspected CAD	LVDD (44), 50%, 65.7 (10.1)	Healthy controls (82), 28.1%, 55.6 (8.9)	LVEDP > 16 mmHg + LVEDVI < 102 mL/m2 + LVEF > 50%
Bruch EU [26]	Cross-sectional	Tei index	IDD	Subjects referred to LHC or known/suspected CAD	HFpEF (29); 24%; 63 ± 9	Normal echo (11)	LVEDP > 16 mmHg + LVEF > 45%
Hayashi Jap [28]	Cross-sectional	Ratios of E wave to peak longitudinal strain (E/LS), E/A and E/E′	LVDD	Subjects who underwent LHC for clinical diagnosis of cardiac diseases	LVEF > 50% (47), of whom 38 with τ ≥ 48 ms and 18 with LVMDP ≥ 12 mmHg	HFrEF (30)	Abnormal LV relaxation = τ ≥ 48 ms; LVMDP ≥ 12 mmHg

*EU* Europe, *AU* Australia, *Jap* Japan, *PH* pulmonary hypertension, *HFpEF* heart failure with preserved ejection fraction, *PAH* pulmonary arterial hypertension, *LVEF* left ventricular ejection fraction, *PCWP* pulmonary capillary wedge pressure, *LVEDP* left ventricular end-diastolic pressure, *NT-proBNP* N-terminal-pro-brain natriuretic peptide, *CAD* coronary artery disease, *RHC* right heart catheterization, *PASP* pulmonary artery systolic pressure, *LHC* left heart catheterization, *LAP* left atrial pressure, *NCD* non-cardiac dyspnoea, *E*′*/A*′ the ratio of early (E′) and late (A′) tissue Doppler diastolic peak velocities, *IVRT* isovolumic relaxation time, *LA* left atrial, *DHF* diastolic heart failure, *E/E′* the ratio of mitral E peak velocity and averaged E′ tissue Doppler angular velocity, *LVH* left ventricular hypertrophy, *RAP* right atrial pressure, *LVDP* left ventricular diastolic pressure, *EDT* E-wave deceleration time, *AR dur-A dur* difference in duration of pulmonary vein flow and mitral flow velocity at atrial contraction, *LAVI* LA volume index, *E/Vp* ratio of mitral E-wave and colour M-mode flow propagation velocity, *LVFP* left ventricular filling pressures, *LVETI* LV ejection time index, *IDD* isolated diastolic dysfunction, *LVEDVI* left ventricular end-diastolic volume index, *LVMDP* left ventricular mean diastolic pressure

## Measures of diagnostic performance: HFpEF

### Multivariable models

In general, multivariable predictors showed good diagnostic performance (Table 2). The highest diagnostic performance was demonstrated by a combination of echocardiography and pulsatile arterial function data with an AUC = 0.95 (95% CI, 0.89–0.98). The addition of aortic pulse pressure to echocardiographic and clinical markers led to a highly significant net reclassification index of up to 33% and reduced the number of undiagnosed HFpEF subjects from 60 to 24 [11]. The H_2_FPEF score showed a very good diagnostic performance to estimate the likelihood of HFpEF among subjects with unexplained dyspnoea [18]. The H_2_FPEF score is based on four clinical characteristics (body mass index, anti-hypertensive medications, atrial fibrillation [AF] and age) and two echocardiographic markers (E/e′ and pulmonary artery systolic pressure) and provided good discrimination of HFpEF from subjects with non-cardiac dyspnoea (NCD) (AUC = 0.84, 0.80–0.88). The performance was maintained in the independent validation cohort with an AUC = 0.87 (0.79–0.94) [18].

**Table 2:** Measures of diagnostic performance of the 20 included studies

Study	Markers	Sensitivity	Specificity	AUC (95% CI) (+ *p* value)	PPV and PNV	Accuracy	LR + and LR-	NRI and IDI
HFpEF studies								
Multivariable models and echocardiographic equations								
Thenappan USA [23]	Age + WHO functional class, hypertension, obesity, DM, CAD, serum creatinine, diuretic, β-blocker, ACE inhibitors/ARBs + LVPWT, LA and RA enlargement			0.935; (0.90–0.97)				
Weber EU [11]	E/e′ + aortic PP + age + ACE-I/ARB + β-blocker + NO-donator	90.09%		0.952 (0.894–0.983 (*p* = 0.0002)				Echo + aortic PP: 32.9%
Cameron USA [24]	E/A, E/e′, LA diameter (1.5 x LA diameter) + (1.7 x E/A) + (1.1 x E/e′ septal)	68%	63%	0.7 (0.62–0.68)	63% and 65%		1.7 and 0.5	
Dokainish USA [17]	1) PASP + LAVI)/2 > 30 2) (E + LAVI)/2 > 57	1) 72% 2) 73%	1) 80% 2) 81%	1) 0.84 (*p* < 0.001) 2) 0.82 (*p* < 0.001)				
Dini EU [16]	CART model (EDT < 150 ms + AR dur-A dur > 30 ms + E/e′ > 13 + LAVI > 40 mL/m^2^ + E/V_p_ > 2)	87%	90%		92% and 84%	88%		
Reddy USA [18]	H2FPEF score: obesity + AF + age > 60 years, treatment with ≥ 2 antihypertensive drugs + E/e′ > 9 + and PASP > 35 mmHg	76%	78%	0.841 (0.798–0.876), *p* < 0.0001			3.49–0.31	
Left ventricular strain and strain rate								
Kasner EU [12]	SR_E_, SR_IVR_, E′/A′, E/SR_E_, E/SR_IVR_ and E/e′ lat			SR_E_ = 0.55, SR_IVR_ = 0.70, e′/A′ = 0.72, E/SR_E_ = 0.75, E/SR_IVR_ = 0.80, E/e′lat = 0.83				
Wang USA [14]	GLS_l_ < − 16%	95%	95%	0.98				
Left atrial strain								
Kurt USA [13]	LA non-invasive stiffness index > 0.99 mmHg	85%	78%	0.85 (0.72–0.98)				
Lundberg, EU [20]	1) Rest LA GS (LA-GS, − 21%) 2) Stress LA GS	1) 93% 2) LA-GS 92%	1) LA-GS 77% 2) LA-GS 88%	1) 0.87 (*p* < 0.001) 2) 0.93 (*p* < 0.001)				
Reddy, 2019 USA [19]	1) LA reservoir strain (< − 24.5%) 2) LA conduit strain (< − 18.4%) 3) LA reservoir strain/E/e′ (< 3) 4) LA reservoir strain/LAVI	1) 56% 2) 64% 3) 65% 4) 58%	1) 94% 2) 63% 3) 78% 4) 85%	1) 0.719 (0.664–0.767), *p* < 0.0001 2) − 0.071 (− 0.102 to − 0.040) (vs reservoir strain), *p* < 0.0001 3) + 0.053 (+ 0.019 to 0.088) vs reservoir strain, *p* = 0.003 4) + 0.032 (+ 0.016 to 0.001) vs reservoir strain, *p* = 0.04				
Singh USA [21]	Peak LA strain (< − 20 mmHg)	71%	92%		83% and 92%	91%		
Telles Au [22]	1) LA global reservoir (< − 32.2%) 2) LA pump strain (< − 15.5%) (AF subjects excluded)	1) 90% 2) 94%	1) 74% 2) 80%	1) 0.85 (0.76–0.95), *p* < 0.001 2) 0.88 (0.77–0.98) *p* < 0.001				1) NRI 12% 2) NRI 14% (vs ESC)
Diastolic stress test markers								
Hammoudi Eu [25]	Ex septal E/é at 25 W ≥ 8	71%	83%	0.79 (0.67–0.92) (*p* < 0.0001)				
Obokata USA [5]	1) ESC + Ex E/e′ > 14 2) ESC + 20 W Ex E/E′ > 14	1) 90% 2) 80%	1) 71% 2) 88%	1) 0.80 (0.68–0.89) (*p* < 0.05 vs ESC) 2) 0.84 (0.73–0.91) (*p* < 0.05 vs ESC)	1) 87% and 77% 2) 93% and 68%		1) 3.1 and 0.1 2) 6.7 and 0.2	
Single conventional echocardiography markers								
Nagueh USA [15]	RAP > 8 mmHg	76%	89%		80% and 87%	85%		
Diastolic dysfunction studies								
Goto Jap [27]	BNP > 22.4 pg/mL + E velocity < 7.4 cm/s	44%	86.8%		61.5% and 76.3%			
Weber EU [29]	LVETI (427.1 ms)	70%	82%	0.81 (0.72–0.89), *p* < 0.0001		76%		
Bruch EU [26]	Tei index > 0.49	37%	86%	0.61 ± 0.08				
Hayashi Jap [28]	E wave/peak longitudinal strain (E/LS) > 680 cm/s	72%	88%	0.80				

The most significant echo markers and multivariable models including echo parameters were reported. *EU* Europe, *AU* Australia, *Jap* Japan, *DM* diabetes mellitus, *CAD* coronary artery disease, *ACE inhibitors* angiotensin-converting enzyme inhibitors, *ARBs* angiotensin II receptor blockers, *LVPWT* left ventricular posterior wall thickness, *LA* left atrial, *RA* right atrial, *RAP* RA pressure, *PASP* pulmonary artery systolic pressure, *CO* cardiac output, *e′* peak early diastolic tissue velocity, *E/e′* peak early filling over early diastolic tissue velocities ratio, *PWV* pulse wave velocity, *PP* pulse pressure, *NO* nitric oxide, *AP* augmented pressure, *Pb* amplitude of the backward wave, *Pf* amplitude of the forward wave, *SR*_*E*_ peak global strain rate (SR) during early diastole, *SR*_*IVR*_ SR during isovolumetric relaxation, *E′/A′* the ratio of early (E′) and late (A′) tissue Doppler diastolic peak velocities, *GLS* global longitudinal strain, *EDT* E-wave deceleration time, *AR dur-A dur* the difference in duration of pulmonary vein flow and mitral flow velocity at atrial contraction, *LAVI* LA volume index, *E/Vp* ratio of mitral E-wave and colour M-mode flow propagation velocity, *BNP* brain natriuretic peptide, *LVFP* left ventricular filling pressures, *Ex* exercise

### Meta-analyses on LA strain

**Fig. 1:**
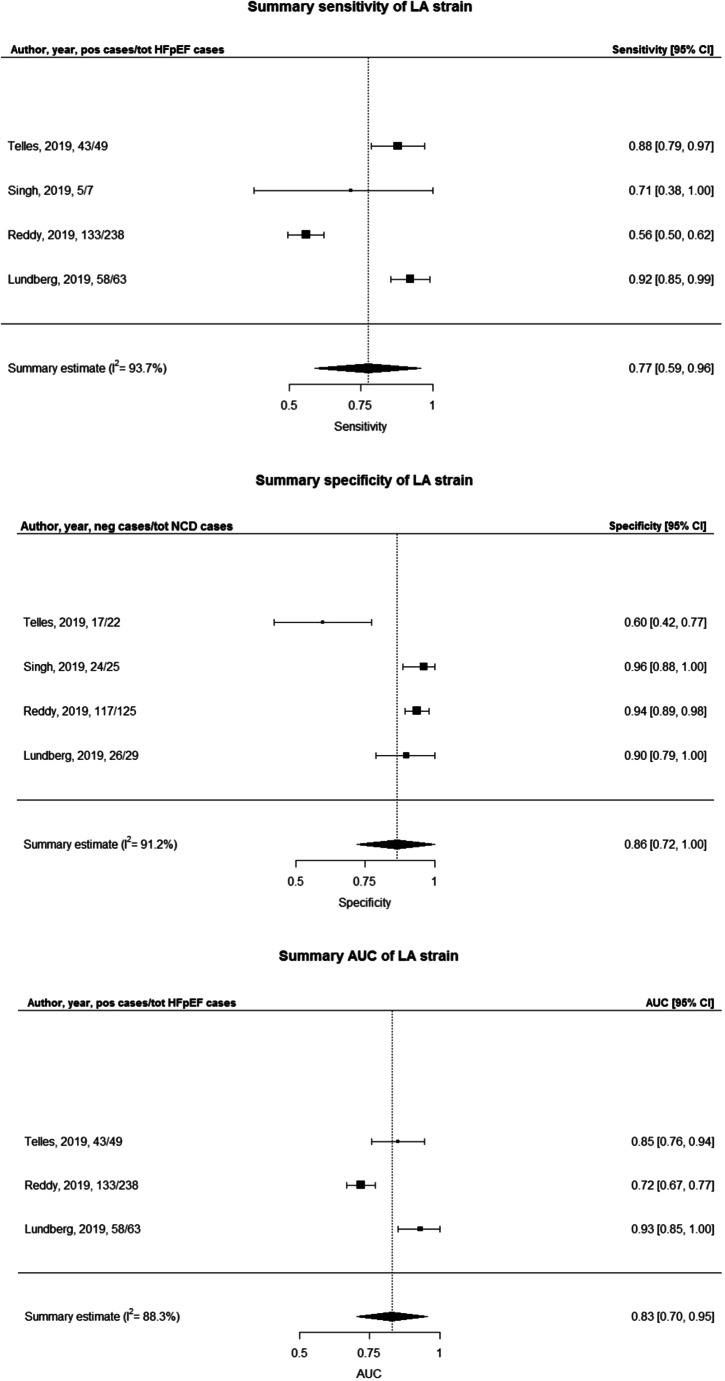
Meta-analysis of sensitivity, specificity and AUC of LA global strain for the detection of HFpEF with controls with non-cardiac dyspnoea

The utilization of LA strain indicated high diagnostic performance without clinical or laboratory data. LA global or reservoir or peak strain was most commonly tested [19–22] with the addition of conduit and booster strain [19, 22] and of indirect measures of LA compliance (LA strain/E/e′) [19] and LA stiffness (E/e′/LA strain) [13, 22]. The best diagnostic ability was demonstrated by LA global strain for detecting elevated LVFP both at rest (AUC = 0.87) and during exercise (AUC = 0.93) in subjects with HF symptoms, outperforming conventional echocardiographic markers such as E/e′ (delta AUC + 0.19 during rest and + 0.37 during stress) and LAVI (delta AUC + 0.08 during rest and + 0.27 during stress) [20]. Four studies reported sensitivity and specificity for LA global strain with a mean of 77% (59–96%; *I*^2^ = 93.7%) and 93% (90–97%; *I*^2^ = 0.22%), respectively, and three studies reported AUCs with a mean of 0.83 (0.70–0.95, *I*^2^ = 88.3) (Fig. 1). The high heterogeneity as shown by the meta-analysis for sensitivity and AUC can be explained by the broad range of values observed among the included studies, which for sensitivity ranged from 56 to 92% and for AUC from 0.72 to 0.93 and by the small sample sizes. On the other hand, all the included studies showed a high ability of LA strain to rule out HFpEF and thus a high specificity with low heterogeneity.

### Diastolic stress test

Two studies evaluated the role of DST in the diagnosis of HFpEF. The first one found that E/e′ at low-level exercise was valuable for predicting abnormal LVFP with a sensitivity of 90% but only in subjects with cardiac disease [25]. The second study evaluated the incremental utility of DST to the diagnostic approaches proposed by ESC and American Society of Echocardiography/European Society of Cardiovascular Imaging (ASE/EACVI) to diagnose HFpEF: the addition of exercise E/e′ to the ESC and ASE/EACVI 2016 proposed algorithm indicated a much higher sensitivity compared with either of them alone (90 versus 60 and 34%, respectively) [5].

### Measures of diagnostic performance: diastolic dysfunction

Five studies investigated echocardiographic markers for the detection of LVDD. The best diagnostic performance was demonstrated by the ratio of E wave to peak longitudinal strain (E/LS) to predict elevated LVFP in a population of subjects with suspected cardiac disease (AUC = 0.86 versus 0.74 of E/e′) [28].

## Discussion

Since HFpEF is the predominant form of HF [1], the detection of this condition gained considerable interest. Standard resting echocardiography has still a pivotal role in the detection of HFpEF, but it provides only indirect evidence of pressure-volume relationships, and it might leave a significant proportion of subjects undetected. In this systematic review, a large variety of echocardiographic markers were investigated and yielded variable results for the diagnostic performance. The main findings are as follows: (1) multivariable models including clinical, echocardiographic and possibly arterial function variables demonstrated the best diagnostic performance. (2) LA strain may provide good discrimination capacity of HFpEF subjects and enhanced diagnostic accuracy beyond conventional echocardiographic measures. (3) Addition of exercise E/e′ to resting echocardiography findings improves HFpEF diagnosis.

### Multivariable models

As expected, multivariable models demonstrated the best diagnostic performance, along the lines of what current guidelines recommend to use in clinical practice for the diagnosis of HFpEF. This can be explained by the complex pathophysiology of HFpEF, which is driven by advanced age and comorbidities, and caused by the interplay of multiple impairments in LV diastolic and systolic function, chronotropic reserve, arterial-ventricular mismatching, vascular and endothelial dysfunction, pulmonary hypertension and impaired systemic vasodilator reserve [30, 31]. Therefore, a multivariable algorithm that provides integrated information on all these aspects is necessary to evaluate diastolic function. Among the included studies, the highest diagnostic accuracy was demonstrated by a multivariable model combining clinical and echocardiographic markers with arterial function measures, thereby demonstrating that measures of pulsatile arterial haemodynamics may complement echocardiography for the diagnosis of HFpEF [11, 31]. Another combination of clinical and echocardiographic markers that provided a better discrimination of HFpEF from NCD than currently used diagnostic algorithms is the H_2_FPEF score, with a delta AUC of + 0.17 (0.12–0.22) in the derivation cohort and a delta AUC of + 0.21 (0.10–0.31) in the test cohort versus 2016 ESC guidelines [18]. However, external validation, which is a crucial step before introducing a new diagnostic model in clinical practice, was not performed. Overall, none of the included studies performed external validation, and only three performed validation in separate groups of subjects belonging to the same research centre [16, 18, 21]. Recently, the H_2_FPEF score was validated in the Alberta HEART population, showing a sensitivity of 90% of a score > 2 to detect HFpEF and a specificity of 82% of a score < 6 to rule out HFpEF [32]. Despite these promising results, the H_2_FPEF score still requires further validation and refinement.

### Left atrial strain

The left atrium plays a key role in HFpEF pathophysiology, and indices of LA mechanics have diagnostic and prognostic utility in HFpEF [33]. STE can assess LA function, remodelling and distensibility, and LA strain can impair independently of LA size [33]. Five recent cross-sectional studies demonstrated the ability of LA strain to correctly classify dyspnoeic subjects as HFpEF with superior sensitivity and specificity than standard echocardiographic parameters [13, 19, 22] or to identify elevated LVFP more accurately than guidelines [20, 21]. Specifically, LA reservoir strain enabled to identify HFpEF from NCD with an AUC = 0.72 (0.66–0.77), outperforming other commonly used indices of diastolic function [19, 20]. Similarly, LA global strain managed to detect elevated LVFP both at rest and during exercise (AUCs = 0.87 and 0.93, respectively) and showed a better agreement with invasively determined LVFP than ESC 2016 guidelines (91 versus 81%) [21]. Among the studies that tested novel indices combining LA strain with Doppler measures of LV pressures, LA non-invasive stiffness showed the highest diagnostic performance in distinguishing subjects with HFpEF from those with LVDD, with an AUC = 0.85 (0.72–0.98) [13]. The meta-analysis of four studies indicated a very high specificity (93%) of LA global strain, in combination with a non-significant heterogeneity (*I*^2^ of approximately 0%) and a good sensitivity (77%) although with consistent heterogeneity (*I*^2^ > 90%), which indicate a high ability of LA strain to rule out HFpEF when normal, and a variable capacity to diagnose HFpEF when abnormal. The meta-analysis of three studies indicated also a good ability of LA strain to predict HFpEF diagnosis with an AUC of 0.83, although with significant heterogeneity (*I*^2^ of 88%). Altogether, these results suggest a potential usefulness of LA strain in the non-invasive diagnostic evaluation of HFpEF. However, the studies that evaluated the diagnostic performance of LA strain established different optimal cut-off values for the identification of HFpEF subjects, ranging from − 32.3 to − 20%, and therefore, further studies are warranted to establish a definitive cut-off for abnormal LA strain. Additionally, it should be noted that STE is not routinely available worldwide and requires post-processing time, which questions its diagnostic utility in clinical practice for non-academic centres.

### Diastolic stress test

Another imaging test with a potential diagnostic role in the diagnosis of HFpEF is the DST. Both ESC and ASE/EACVI guidelines already recommended to perform DST when resting echocardiography does not explain the symptoms of HF, especially when dyspnoea is present only with exertion [2, 3]. Recently, the DST has been integrated in the new HFA diagnostic recommendations, as part of the advanced HFpEF workup, to be performed if a subject who already underwent clinical, biomarkers and resting echocardiography assessment has an intermediate HFA-PEFF score [7]. The utility of exercise data is clearly evident on top of resting echocardiographic data, as the utilization of exercise E/e′ alone (> 14) indeed significantly improved the sensitivity of the diagnostic work-up to 90% compared with 60% of ESC guidelines [5]. Addition of exercise E/e′ also improved classification beyond the resting ESC criteria, with a negative predictive value of 87 versus 83% [5]. Hence, our results confirm the utility of DST not only to identify HFpEF in euvolemic subjects with inconclusive resting echocardiography but also to rule out HFpEF, when unequivocally normal. However, we must point out that the feasibility and the quality of echocardiographic measures decrease during exercise; for instance, tricuspid regurgitation velocity was measureable only in 49% of subjects at peak exercise [5]. Moreover, although a low-level exercise test with stepwise increase of the workload is recommended for the DST, there is no universally adopted protocol at the moment.

### Strengths and limitations

To our knowledge, this is the first systematic review on novel echocardiographic markers for HFpEF and LVDD including a meta-analysis. Multiple databases were extensively searched, and article selection, data extraction and quality assessment were performed in duplicate according to a standardized protocol. Moreover, no geographical differences were detected, which increases the generalizability of the results. The review findings were limited by the heterogeneity and the quality of the included studies, which applied to the study design (case control versus cross-sectional), the study population (subjects with unexplained dyspnoea versus subjects with suspected coronary artery diseases), the reference standard (different invasive measures with different cut-off values) and the index test (different combinations of echocardiographic techniques and clinical markers). In addition, quality assessment showed a large number of studies with high risk of bias across several domains. For example, 15 of 20 studies excluded subjects not in sinus rhythm. It is well known that HFpEF with concurrent arrhythmias and especially with AF is increasingly common [34]. The exclusion of subjects with AF questions the possibility to efficiently and practically use the newly tested echocardiographic markers in individuals with HFpEF and rhythm abnormalities, limiting generalizability. Another aspect that may have affected the results is the interpretation of the index test, since the echocardiographic analysis was often not blinded from the catheterization or not simultaneous, performed by different investigators, and only in three studies, the cut-off value of the echocardiographic marker was specified before the analysis  [11, 22, 24, 25]. This could have resulted in an overestimation of performance of the proposed predictor, questioning its validity.

## Conclusions

In conclusion, despite the considerable heterogeneity of the included studies which does not allow to draw definite conclusion, this study supports an integrated approach for the diagnosis of HFpEF, which includes multiple clinical and echocardiographic measures. New echocardiographic indices such as LA strain and DST data have potential diagnostic value to enhance the detection of HFpEF and LVDD. However, before their implementation into the diagnostic workup, their added diagnostic utility, beyond the established clinical and echocardiographic HFpEF features, should be proven by larger studies of HFpEF versus NCD subjects.

## Electronic supplementary material

ESM 1 (DOCX 12 kb).

ESM 2 (JPG 125 kb).

ESM 3 (JPG 141 kb)

ESM 4 (DOCX 22 kb).

## List of abbreviations

HFpEF: Heart failure with preserved ejection fraction
LVEF: Left ventricular ejection fraction
NPs: Natriuretic peptides
LAVI: Left atrial volume index
LVMI: Left ventricular mass index
LVDD: Left ventricular diastolic dysfunction
LVFP: Left ventricular filling pressures
HFrEF: Heart failure with reduced ejection fraction
STE: 2-D speckle tracking echocardiography
DST: Diastolic stress test
QUADAS: Quality Assessment of Diagnostic Accuracy Studies
AUC: Area under the curve
PH: Pulmonary hypertension
NCD: Non-cardiac dyspnoea
AF: Atrial fibrillation
HFA: Heart Failure Association
ESC: European Society of Cardiology
ASE/EACVI: American Society of Echocardiography/European Association of Cardiovascular Imaging
