# Supplementary material for: Diagnostic value of echocardiographic markers for diastolic dysfunction and heart failure with preserved ejection fraction
Source: Heart Fail Rev. 2020 Jun 2;27(1):207–18. doi: 10.1007/s10741-020-09985-1 (PMC8739319; doi:10.1007/s10741-020-09985-1)
Supplement: Supplementary file 1 — (DOCX 12 kb). [file 10741_2020_9985_MOESM1_ESM.docx]

**Diagnostic Value of Echocardiographic Markers for Diastolic Dysfunction and Heart Failure with Preserved Ejection Fraction.**

**Authors disclosures**

Dr. Dal Canto, Dr. Remmelzwaal, Dr. van Ballegooijen, Dr. Handoko, Prof. Heymans, Dr. van Empel, Prof. Paulus, Prof. Nijpels, Dr. Elders and Prof Beulens have no conflicts of interest or financial ties to disclose.
