# Supplementary figures and images for: Diagnostic value of echocardiographic markers for diastolic dysfunction and heart failure with preserved ejection fraction
Source: Heart Fail Rev. 2020 Jun 2;27(1):207–18. doi: 10.1007/s10741-020-09985-1 (PMC8739319; doi:10.1007/s10741-020-09985-1)

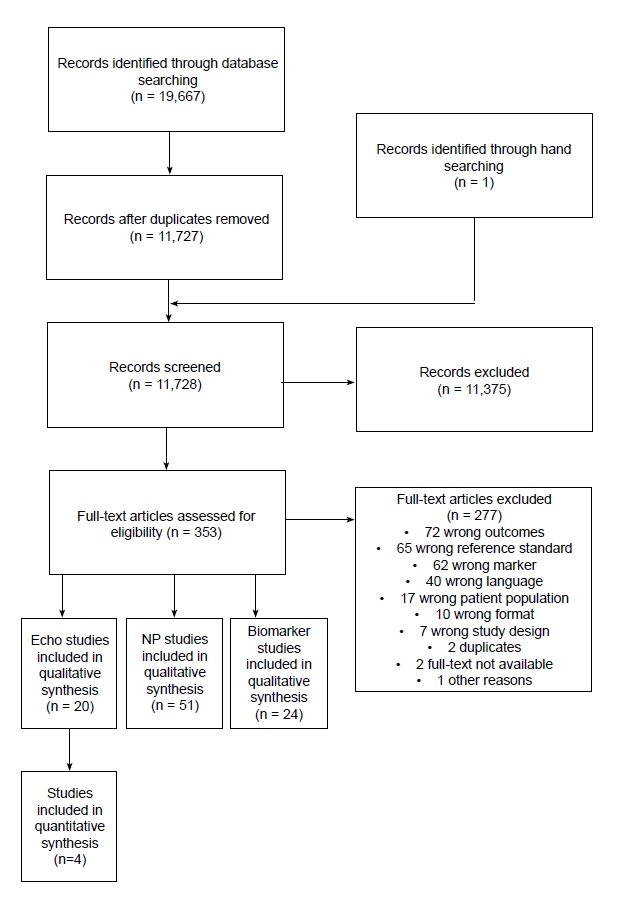

Supplement: Supplementary file 2 — (JPG 125 kb). [file 10741_2020_9985_MOESM2_ESM.jpg]

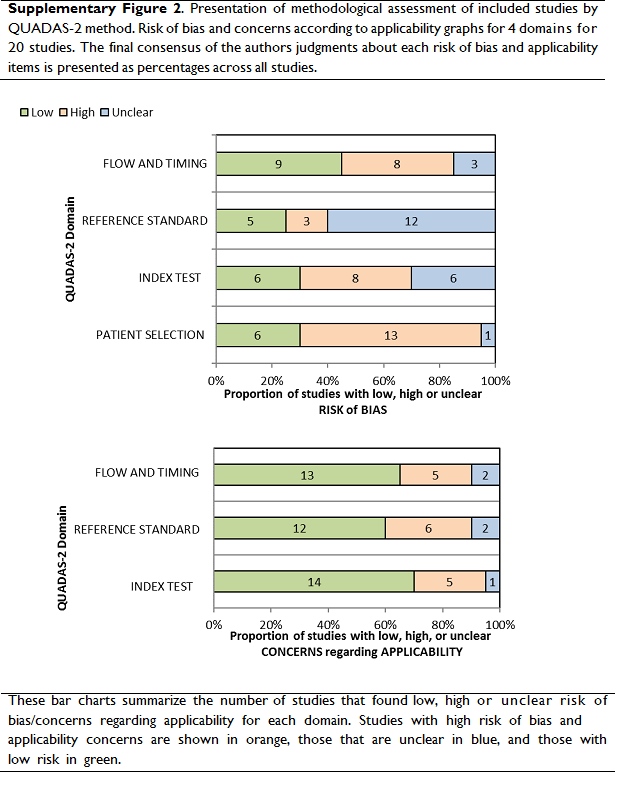

Supplement: Supplementary file 3 — (JPG 141 kb) [file 10741_2020_9985_MOESM3_ESM.jpg]
