# Supplementary material for: Diagnostic value of echocardiographic markers for diastolic dysfunction and heart failure with preserved ejection fraction
Source: Heart Fail Rev. 2020 Jun 2;27(1):207–18. doi: 10.1007/s10741-020-09985-1 (PMC8739319; doi:10.1007/s10741-020-09985-1)
Supplement: Supplementary file 4 — (DOCX 22 kb). [file 10741_2020_9985_MOESM4_ESM.docx]

**Diagnostic Value of Echocardiographic Markers for Diastolic Dysfunction and Heart Failure with Preserved Ejection Fraction.**

**Supplementary Table 1**

| **Supplementary table 1.**Studies that met all criteria were included in the review. |
| --- |
| ***Inclusion criteria:*** |
| 1. Having LVDD or HFpEF as outcome defined according to current recommendations (2) |
| 1. Testing the diagnostic value of novel indices of LVDD, used alone or in combination with clinical/laboratory markers or included in multivariable models/equations. |
| 1. Reporting a diagnostic performance measure (area under the receiver operating curve (AUC), sensitivity, specificity, positive predictive value (PPV), negative predictive value (NPV), positive likelihood ratio test (LR+), negative likelihood ratio test (LR-), prediction accuracy, integrated discrimination improvement (IDI) or net reclassification improvement (NRI)). |
| 1. Use of a control population of either healthy controls or subjects with  HFrEF. |
| 1. Testing the performance of the diagnostic marker in a cross-sectional study design (with a maximum follow-up time of 2 years) |
| 1. Written in English or Dutch. |
| ***Exclusion criteria*** |
| 1. Studying the performance of the diagnostic marker in patient groups with rare diseases (e.g. Beta thalassemia). |
| 1. Studying the performance of the diagnostic marker in a prospective study with a follow-up time no longer than 2 years. |
| 1. Testing the diagnostic performance of single echocardiographic parameters or combinations of parameters already included in the currently proposed algorithms for the determination of LVDD (2, 3). |
| 1. Testing the performance of the diagnostic marker for the detection of acute HF. |
| 1. Studies performed on children and studies performed on animals. |
| 1. Review, systematic review, meta-analysis, editorial or conference abstract and randomized clinical trials (RCT) studies. |
